# Assessing dietary intake among infants and toddlers 0–24 months of age in Baltimore, Maryland, USA

**DOI:** 10.1186/1475-2891-12-52

**Published:** 2013-04-26

**Authors:** Sangita Sharma, Fariba Kolahdooz, Lauren Butler, Nadine Budd, Berenice Rushovich, Galina L Mukhina, Joel Gittelsohn, Benjamin Caballero

**Affiliations:** 1 Department of Medicine, University of Alberta, 5-10 University Terrace, 8303 112 Street, Edmonton AB T6G 2T4, Canada; 2 Center for Human Nutrition, Bloomberg School of Public Health, Johns Hopkins University, Baltimore, MD, USA; 3 School of Social Work, University of Maryland, Baltimore, MD, USA; 4 School of Medicine, Johns Hopkins University, Baltimore, MD, USA

**Keywords:** Children under two, Dietary assessment, Dietary recalls, Food frequency questionnaire, Growing leaps and bounds program

## Abstract

**Objective:**

To characterize food and nutrient intake and develop a population-specific food list to be used as a comprehensive dietary assessment tool for Baltimore infants and toddlers aged 0–24 months. The data were used to inform the Growing Leaps and Bounds (GLB) program, which promotes early obesity prevention among Baltimore infants and toddlers.

**Research methods & procedures:**

A cross-sectional dietary survey using 24-hour recalls among randomly selected primary caregivers of infants and toddlers was conducted.

**Results:**

Data were collected from 84 children, (response rate 61%) 45 boys; 39 girls. Mean daily energy intakes were 677 kcal, 988 kcal, and 1,123 kcal for children 0–6 months, 7–12 months and 13–24 months, respectively. Infants 0–6 months had higher percentage of energy from fat (48%) than infants 7–12 months (34%) and 13–24 months (31%). Mean daily intakes for all nutrients among 0–12 months old were ≥ Dietary Reference Intakes (DRI), while toddlers 13–24 months had inadequate vitamins A, D, and E intake. Breastfeeding occurred in 33% of infants and toddlers 0 to 6 months, while less than 3% of those aged 7 to 24 months were breastfed. A 104-item food list with eight food and drink categories was developed.

**Conclusions:**

Infants were formula fed with a higher frequency than they were breastfed. The consumption of high-sugar and high-fat foods (e.g. sweetened drinks, French fries) increased with each age group, which can increase the risk of childhood obesity.

## Background

Ten percent of infants in the US are at or above the 95^th^ percentile for weight [1]. Overweight toddlers are more likely to grow up as overweight adults, a condition that carries elevated risks for co-morbidities, such as cardiovascular disease, diabetes, and cancer [1-6]. According to the 2007–2008 National Health and Nutrition Examination Survey (NHANES), from 1980 to 2008, obesity doubled (5.0% to 10.4%) among children aged 2–5 years, tripled (6.5% to 19.6%) in children aged 6–11 years, and almost quadrupled (5.0% to 18.1%) in adolescents aged 12–19 years [1]. The Youth Risk Behavior Survey found that 18.5% of adolescents in Baltimore, MD, had BMIs greater than the 95^th^ percentile, as compared to 12.0% for adolescents across the 39 included states [7], indicating the need for a comprehensive dietary assessment that can be used to monitor dietary inadequacy among Baltimore infants and children.

Comprehensive dietary assessment among infants and toddlers is essential for monitoring nutrients and energy requirements needed for growth and development [8,9]. Grummer & Strawn (2008) found infants who were fed complementary foods were more likely to discontinue breastfeeding and consume high-sugar, high-fat foods within the first year of life, compared to those who were not [10]. Factors during infancy and early childhood, such as inappropriate introduction of complementary foods and energy imbalance have been linked to the increasing prevalence of childhood obesity [2,10-12]. Findings from the Feeding Infants and Toddlers Study (FITS) indicate that among US infants and toddlers, 33% are consuming diets lacking in fruits and vegetables and nearly half of infants age 7–8 months are consuming sweet drinks or desserts [13]. Consequently, US infants are exceeding energy requirements and toddlers are not meeting the fiber recommendations [14].

Most dietary assessments of infants and toddlers (0–24 months) have used repeated 24-hour dietary recalls, weighed food diaries, or multi-day food records (i.e. NHANES, Nationwide Food Consumption Survey (NFCS), FITS) [13,15,16]. 24-hour recalls have proven valuable to develop comprehensive methods of dietary assessment including population-specific Food Frequency Questionnaires (FFQ) [17-21]. To our knowledge, there is no comprehensive dietary assessment tool for African-American infants and toddlers in Baltimore.

This study aimed to characterize food and nutrient intake among infants and toddlers 0–24 months in Baltimore and to develop a population-specific FFQ with an inclusive food list identifying items that contribute substantially to their nutrient intake.

## Methods

### Sampling

Participants were primary caregivers of randomly selected infants and toddlers from the GLB study in Baltimore City, Maryland, 138 caregivers were familiarized with the purpose of this study and asked to participate by completing a 24-hour dietary recall. Inclusion criteria specified that children, at birth, weighed > 1500 g, did not require specialized care, and were discharged 5 days after birth. Participants were recruited from two John Hopkins Community Physicians (JHCP) centers, and two grocery stores, one located within Baltimore City and one located in Baltimore County. All participants signed a consent form, and were compensated with a gift certificate ($10 value) to Target or Wal-Mart. The study was approved by the Johns Hopkins Bloomberg School of Public Health Committee on Human Research.

### Data collection

Between January and February 2008, single 24-hour dietary recalls were collected from primary caregivers and were asked by a trained researcher to recall all foods and drinks consumed by their children in the previous 24 hours. Using the same approach as previous studies [19-21], food models (NASCO Company, 901 Jamesville Ave, Fort Atkinson, Wisconsin 53538), containers of common baby foods, and household utensils (e.g. bowls, cups, and spoons) were used to estimate amounts consumed. The participants were asked to report multiples or portions of food servings (e.g. half a 120 ml jar). An additional list of questions prompted primary caregivers about easily forgotten foods, such as snacks and drinks and asked about special dietary practices (e.g. lactose-free diet). Recalls were (1) recorded by trained interviewers, (2) included weekdays and weekends, and (3) standardized dietary assessment forms were utilized. All data was examined by the project coordinator to ensure completeness prior to the end of each interview.

All food portions reported on the dietary recalls were weighed using electronic Salter kitchen scales (Aquatronic Baker’s Dream Scale, Salter Houseware, Ltd., Tonbridge, Kent, UK). Portion size was defined as the amount of the food served in one sitting [22].

Only frequency of breastfeeding was recorded due to the difficulty of approximating amounts consumed during breastfeeding. Instead, amounts consumed were estimated based on published literature (see data analysis) [14,23].

### Data analyses

Infants and toddlers >24 months of age and with extreme energy intake (outside the range of mean ± 2 Standard Deviation (SD)) were excluded from analyses as outliers. All foods and drinks reported were tabulated to determine the frequency of consumption.

Breastfed infants 0–6 months of age were assigned 780 ml of breast milk per day. For infants in this age range who consumed both breast milk and formula, the volume of formula was subtracted from 780 ml to obtain the quantity of breast milk consumed. For breastfed infants ≥ 7 months, 600 ml was entered for total daily breast milk intake and those consuming both breast milk and formula had the volume of formula subtracted from the amount of recommended breast milk to estimate the volume of breast milk consumed [14].

The 24-hour recalls were coded by assigning weights (grams) to all portions reported and were entered manually into Nutribase Manager version 9 (Cybersoft Inc., Phoenix, AZ, USA), a dietary database based on the US Department of Agriculture National Nutrient Database for Standard Reference. Mean daily energy and nutrient intake were analyzed using Nutribase software. Dietary adequacy was determined using the Estimated Average Requirements (EAR) for infants and children per Dietary Reference Intake (DRI) age group (0–6 months, 7–12 months, and 1–3 years) [24]. If the EAR was not available, as for fiber, vitamins A, D, and E for infants under the age of 12 months, Adequate Intake (AI) was used. Additional data analyses were performed using SAS version 9.3 (Cary, NC. SAS Institute Inc).

### Generating the food list

Every food or drink consumed by at least two children were included in the food list and used to develop a draft FFQ. Blank lines were added to the draft FFQ to capture any omitted food or drink, and participants were asked to recall any foods consumed that were not listed. Information on feeding practices of the primary caregiver was obtained by including additional questions. Portion sizes were not listed on the draft FFQ because of the difficulty in collecting accurate portion size.

The draft FFQ was pilot-tested among a convenience sample of 16 participants recruited from three of the four centers that represented all age groups and both sexes to identify foods that were not reported in the recalls. Results were used to generate a final food list.

## Results

Among 138 caregivers, 84 (61%) completed 24-hour dietary recalls. Seven toddlers were excluded due to age (>24 months), and four infants who had extreme energy intake (≥ 1857 kcal) were also excluded, leaving 73 caregivers. Several reasons were noted for non-participation, including parental refusal, illness of the child, and insufficient knowledge of the foods eaten by the child (i.e. if the child was with another caregiver). Demographic information from the 73 infants and toddlers were also recorded. Approximately 63% of respondents were African American, 29% were Caucasian, 7% were of mixed ethnicity, and 1% reported other/unknown ethnicity. Data is presented by the following age categories: 0–6 months (n = 21, mean age = 4.2 mos), 7–12 months (n = 20, mean age = 9.3 mos), and 13–24 months (n = 32, mean age = 18.1 mos). Sixteen of the infants and toddlers took supplements; six took Flintstone vitamins, two took Gummy vitamins, and eight took liquid drops (i.e. liquid iron). Of breastfed babies under six months of age, three used liquid supplement drops, and one used vitamin D drops. One toddler in the 13–24 months group was given Echinacea and vitamin C.

### Dietary intake

Table 1 presents the mean intakes of energy, macronutrients, and micronutrients, with the reference intakes for each age group included as a reference. The mean (and SD) of daily energy intakes were 677 ± 226 kcal, 988 ± 280 kcal, and 1,123 ± 286 kcal for infants 0–6 months, 7–12 months and toddlers 13–24 months, respectively. Mean percentage of energy from protein was 9%, 10%, and 13% for infants 0–6 months, 7–12 months, and toddlers 13–24 months, respectively. Infants 0–6 months had higher percentage of energy from fat (48%) than the two older age groups (34% for 7–12 months and 31% for 13–24 months).

**Table 1:** Mean daily intake of energy and selected nutrients of Baltimore infants and toddlers

	**All ages (n = 73)**	**0 - 6 months (n = 21)**		**7 - 12 months (n =20)**		**13 - 24 months (n = 32)**	
	**Mean (SD)**	**Mean (SD)**	**RDA/AI**^**1**^	**Mean (SD)**	**RDA/AI**^**1**^	**Mean (SD)**	**RDA/AI**^**1**^
Age (months)	11.7 (6.6)	4.2 (1.1)	-	9.3 (1.7)	-	18.1 (3.5)	-
Energy (kcal)	957.3 (327.4)	676.5 (225.7)	-	987.6 (280.1)	-	1122.7 (285.8)	-
Protein (g)	28 (16.6)	14.5 (5.7)	9.1^*2*^	25.4 (12.5)	11^*4*^	38.6 (16.5)	13^*4*^
Carbohydrate (g)	128.2 (51.3)	75.1 (32.5)	60^*2*^	139.7 (36.3)	95^*2*^	155.8 (41.9)	130^*4*^
Fat (g)	38.1 (14.0)	35 (9.0)	31^*2*^	38.6 (12.3)	30^*2*^	39.9 (17.1)	-
% energy from protein	10.9 (3.6)	8.5 (1.8)	-	9.7 (2.8)	-	13.3 (3.6)	5-20^*3*^
% energy from carbohydrates	52.5 (9.7)	43.9 (3.9)	-	56 (5.3)	-	56 (11.0)	45-65^*3*^
% energy from fat	36.6 (9.8)	47.6 (4.8)	-	34.3 (4.0)	-	30.8 (8.8)	30-40^3^
Sugars (g)	76.4 (30.9)	59.8 (29.2)	-	81.5 (26.7)	-	84.1 (30.2)	< 25%^*3*^
Dietary fiber (g)	4.8 (4.2)	1 (1.8)	-	4.7 (3.3)	-	7.4 (3.8)	19^*2*^
Saturated fat (g)	14.2 (6.4)	13.1 (4.4)	-	14 (5.0)	-	15.1 (8.0)	low as possible^*3*^
Monounsaturated fat (g)	11.3 (5.7)	12 (4.4)	-	11.8 (4.4)	-	10.4 (6.9)	-
Polyunsaturated fat (g)	5.7 (3.2)	6.5 (3.0)	-	7.4 (2.6)	-	4.3 (3.0)	-
Omega-3 fatty acid (g)	0.5 (0.3)	0.6 (0.3)	0.5^*2*^	0.7 (0.3)	0.5^*2*^	0.4 (0.3)	0.7^*2*^
Omega-6 fatty acid (g)	4.5 (2.9)	5.7 (2.7)	4.4^*2*^	6.3 (2.5)	4.6^*2*^	2.6 (1.8)	7^*2*^
Vitamin A (μg-RAE)	419.3 (246.5)	540 (212.7)	400^*2*^	509.3 (203.5)	500^*2*^	284 (223.0)	300^*4*^
Thiamin (mg)	0.8 (0.6)	0.7 (0.5)	0.2^*2*^	1.2 (0.7)	0.3^*2*^	0.7 (0.5)	0.5^*4*^
Riboflavin (mg)	1.2 (0.8)	0.9 (0.6)	0.3^*2*^	1.5 (0.7)	0.4^*2*^	1.2 (0.9)	0.5^*4*^
Niacin (mg)	10.1 (7.2)	8.8 (6.1)	2^*2*^	14.7 (8.6)	4^*2*^	8 (5.4)	6^*5*^
Vitamin B-6 (mg)	0.7 (0.5)	0.4 (0.3)	0.1^*2*^	0.7 (0.4)	0.3^*2*^	0.8 (0.6)	0.5^*4*^
Total folate (μg-DFE)	170.8 (145.3)	132.0 (76.4)	65^*2*^	170.4 (74.3)	80^*2*^	196.6 (201.8)	150^*4*^
Vitamin B-12 (μg)	2 (1.6)	1.6 (0.8)	0.4^*2*^	1.8 (1.0)	0.5^*2*^	2.5 (2.2)	0.9^*4*^
Vitamin C (mg)	124.5 (95.8)	75 (53.3)	40^*2*^	157.5 (111.4)	50^*2*^	136.4 (94.3)	15^*4*^
Vitamin D(μg)^*5*^	5.1 (4.0)	6.3 (5.0)	10^*2*^	5.9 (3.5)	10^*2*^	3.8 (3.1)	15^*4*^
Vitamin E (mg)^*6*^	6 (5.8)	8.6 (6.3)	4^*2*^	9.7 (4.7)	5^*2*^	2.1 (2.9)	6^*4*^
Calcium (mg)	622.9 (339.2)	556.8 (257.7)	200^*2*^	711.5 (325.9)	260^*2*^	610.9 (381.6)	700^*4*^
Magnesium (mg)	87.6 (51.7)	59.6 (32.1)	30^*2*^	108.9 (50.3)	75^*2*^	92.7 (55.0)	80^*4*^
Potassium (mg)	1,045.8 (549.2)	730.3 (340.2)	400^*2*^	1,092.6 (358.6)	700^*2*^	1,223.6 (661.6)	3000^*2*^
Sodium (mg)	988.5 (868.4)	199.7 (60.6)	120^*2*^	794.8 (659.8)	370^*2*^	1,627.2 (775.4)	1000^*2*^
Iron (mg)	12.9 (10.6)	12.2 (9.3)	0.27^*2*^	21.1 (12.6)	11^*4*^	8.2 (5.7)	7^*4*^
Zinc (mg)	4.8 (3.1)	4.7 (2.3)	2^*2*^	5.4 (2.3)	3^*4*^	4.5 (3.9)	3^*4*^

RAE, retinol activity equivalents; DFE, dietary folate equivalents.

^1^The Dietary Reference Intakes are presented in this table using Adequate Intakes or Recommended Dietary Allowances for age groups 0–6 months, 7–12 months and 1–3 years.

^2^Adequate Intake.

^3^Acceptable Macronutrient Distribution Range.

^4^Recommended Dietary Allowance.

^5^As cholecalciferol. In the absence of adequate exposure to sunlight.

^6^As alpha–tocopherol.

Within the 0–6 months and 7–12 months age groups, the mean intake of all nutrients met the dietary recommendations. However the 13–24 months age group had inadequate mean intake of fiber, omega-3 and omega-6 fatty acids, potassium and vitamins A, D and E, while the mean intake for vitamin C and calcium exceeded daily recommendations (Table 1).

Table 2 presents the frequencies of food groups consumed (times/day). Formula was the most frequently reported food consumed by infants 0–6 and 7–12 months and was consumed more than four times/day by more than 85% of these groups. Breast milk was only consumed 1.7 times/day by 33% of infants aged 0–6 months. Only 14% of infants 0–6 months were exclusively breastfed on the day of the recall (data not shown).

**Table 2:** Frequencies of food consumption in Baltimore infants and toddlers

**Food and nutrients intake**	**Frequency of Intake (% of children reporting)**		
	**0-6 months**^**1**^	**7-12 months**^**1**^	**13-24 months**^**1**^
	**(n = 21)**	**(n = 20)**	**(n = 32)**
**Food group**			
Formula^2^	4.4 (85.7)	3.7 (90.0)	--
Cereals	1.7 (57.1)	2.0 (85.0)	1.1 (78.1)
Breast milk	1.7 (33.3)	--	0.6 (3.1)
Vegetables^3^	0.2 (23.8)	0.6 (50.0)	0.8 (53.1)
Unsweetened drinks^4^	0.1 (14.3)	1.5 (70.0)	2.1 (87.5)
Baby food-vegetables and main dishes	0.2 (9.5)	0.9 (60.0)	0.2 (9.4)
Potatoes	0.2 (9.5)	0.3 (30.0)	0.4 (31.3)
Breads and crackers	0.05 (4.8)	0.5 (35.0)	0.8 (65.6)
Dairy products and eggs	0.05 (4.8)	0.6 (40.0)	2.4 (90.6)
Fruits^5^	0.4 (23.8)	1.2 (90.0)	0.7 (43.8)
Sweetened drinks^6^	--	0.2 (15.0)	0.7 (50.0)
Meat and chicken	--	0.7 (45.0)	1.5 (84.4)
Rice, pasta, pizza	--	0.6 (35.0)	1.3 (90.6)
Snacks and desserts	--	1.3 (80.0)	1.8 (100.0)

^1^Institute of Medicine of the National Academies, 2005; Joint WHO/FAO Expert Consultation, 2005.

^2^Includes mixed with cereal.

^3^Excludes potatoes.

^4^Includes water, water with 100% juice, 100% and infant brand 100% juice, diet drinks.

^5^Includes apple sauce, baby food fruits.

^6^Includes sweet tea, sodas, juice drinks (not 100% juice).

Consumption of dairy products rose steadily with age, and at 13 months, around 90% of all toddlers were consuming dairy products 2.4 times/day, coinciding with elimination of formula feeding. The consumption of high-sugar and high-fat foods (i.e. sweetened drinks, chips) increased with each age group. Almost all potatoes consumed by toddlers aged 19 months or older were french fries and consumption increased steadily with age (data not shown). Sweetened drinks, including sodas and fruit drinks, were consumed 0.7 time/day by 50% of toddlers after the age of 13 months and all toddlers in this group had a snack (i.e. chips, cookies) or dessert 1.8 times/day. The percentage of toddlers 13–24 months consuming fruit decreased from half to 44% when compared to infants 7–12 months. Overall, the five most frequently reported individual food items among infants and toddlers 0–24 months were; formula, cow’s milk, apple juice, chicken, and rice cereal (data not shown).

### Food sources of energy and nutrients

As seen in Table 3, formula was the top contributor of energy, fat, sugar and protein for infants 0–6 and 7–12 months of age with breast milk as the second highest contributor of energy, fat, sugar, and protein among infants 0–6 months of age. Among infants 7–12 months old oatmeal, cereal, or other porridges was the second highest contributor to energy. Among toddlers 13–24 months old milk, noodles and pasta, and unsweetened drinks were the top three energy sources.

**Table 3:** Main foods contributing (%) to energy, fat, protein and sugar among Baltimore infants and toddlers

**Food**	**% Energy**	**Food**	**% Fat**	**Food**	**% Protein**	**Food**	**% Sugar**
**Age 0–6 months**							
Formula	71.1	Formula	73.1	Formula	74.5	Formula	70.6
Breast milk	20.3	Breast milk	24.6	Breast milk	13.9	Breast milk	22.6
Oat, rice cereal, porridge	4.0	Oat, rice cereal, porridge	1.1	Oatmeal, rice cereal, porridge	4.8	Baby food fruit	3.6
Baby food fruit	1.7	Yogurt	0.6	Baby food & mixed dishes	3.0	Unsweetened drinks	1.9
Yogurt	0.8	Unsweetened drinks	0.5	Baby food vegetables	1.4	Yogurt	1.3
Baby food vegetables	0.7	Crackers	0.3	Yogurt	1.3	Baby food vegetables	0.7
Unsweetened drinks	0.7	Baby food vegetables	0.1	Baby food fruit	0.6	Baby food & mixed dishes	0.1
Baby food & mixed dishes	0.6	Potatoes	0.1	Crackers	0.2	Oat, rice cereal, porridge	0.1
**Age 7–12 months**							
Formula	46.5	Formula	61.1	Formula	39.5	Formula	52.8
Oatmeal, rice cereal, porridge	8.8	Butter	5.6	Chicken and turkey	11.5	Unsweetened drinks	20.5
Unsweetened drinks	8.1	Baby food & mixed dishes	4.0	Baby food & mixed dishes	11.1	Baby food fruit	5.2
Baby food & mixed dishes	5.4	Milk	3.9	Oat, rice cereal, porridge	6.8	Sweetened drinks	4.8
Cookies	2.9	Egg dishes	3.5	Milk	5.8	Oat, rice cereal, porridge	3.5
Milk	2.8	Processed meats	3.2	Egg dishes	5.6	Milk	3.0
Baby food fruit	2.6	Oat, rice cereal, porridge	2.6	Rice	3.5	Cookies	2.6
**Age 13–24 months**							
Milk	17.1	Milk	25.4	Milk	26.6	Unsweetened drinks	25.7
Noodles and pasta	11.4	Chicken and turkey	9.0	Chicken	21.7	Sweetened drinks	20.4
Unsweetened drinks	9.0	Noodles and pasta	7.7	Noodles and pasta	12.1	Milk	20.3
Chicken	7.2	Egg dishes	6.0	Beef or pork	6.5	Fresh fruit	4.4
Sweetened drinks	6.5	Butter	5.6	Egg dishes	5.3	Candy and chocolate	2.8
Vegetables	3.0	Processed meats	5.4	Processed meats	3.4	Sweetened cereal	1.9
Egg dishes	2.8	Chips	4.4	Vegetables	2.7	Noodles and pasta	1.9

### The final food list

The final food list contains 104 foods and drinks including: breast milk, formula, 8 dairy products, 10 drinks, 5 cereals, 15 fruits, 4 baby food dishes, 16 vegetable-based items, including tofu and beans (not including those in soup or stew), 12 rice, pasta, and pizza items, 13 meat dishes, 8 bread and cracker items, and 11 snack and dessert items. An additional line for multivitamin usage, such as Flintstones, is included. Five questions on feeding practices are included in the final version to strengthen participants’ recall: ‘Do you usually add cereal to the bottle/cup?; Do you usually add milk to oatmeal/cereal?; Do you add sugar to cereal?; Is your pasta usually whole wheat?; Do you add butter/margarine to rice/pasta/vegetables?’. Similar to previous studies [20,21], the food list was developed to capture all foods and drinks that contribute to more than 85% of the nutrients, therefore, foods such as condiments that contribute small percentages of energy/nutrients to the diet were excluded. Foods not listed in the initial list but added to the final food list included melon/cantaloupe, orzo, and lasagna. Additional file 1 describes the foods and drinks included in the final food list and Additional file 2 contains a sample page of the FFQ.

## Discussion

This study presents data on the dietary intake and food patterns of infants and toddlers specific to Baltimore, MD. Devaney et al., (2004) found that US toddlers aged 12–24 months had similar mean daily energy intakes to toddlers 13–24 months in this Baltimore population (1,249 kcal and 1,123 kcal, respectively) [14]. Among our study population, the mean intakes of all nutrients examined for infants 0–6 and 7–12 months of age were, on average, adequate. However toddlers 13–24 months of age had inadequate mean intakes of omega-3 and omega-6 fatty acids, fiber, vitamins A, D, and E.

The consumption of high-sugar, high-fat foods increased with each age group; comparable observations have been reported in FITS [13]. Similarly, data from the 2005–2007 Infant Feeding Practices Study II (IFPS II) found that at 12 months two-thirds of infants were consuming high sugar and fat foods, including french fries, or sweetened drinks, and 77% of infants were consuming fruit juice [10]. Similarly we found that on a daily basis most toddlers consumed a dessert or snack and fruit juice more than once, and half consumed a sweetened drink.

For this study, a food list was compiled from the foods reported in the 24-hour dietary recalls based on our study population’s current eating habits, similar to previous studies [20,21,25]. The final food list is essential in the development of an accurate dietary assessment tool (i.e. FFQ) that can be used in intervention strategies for the GLB obesity prevention program [26,27]. For example, formula feeding was more frequent than breastfeeding in our study population. Research indicates that a shortened period of breastfeeding increases the risk of childhood obesity [28,29]. Furthermore, formula-fed newborns experience weight gain, which can increase the risk of childhood chronic disease [30-32]. Other strategies to consider for nutritional intervention include reducing milk and sweetened drinks and increasing fruit, vegetable, and whole grain intake among toddlers 13–24 months. Milk and sweetened drinks were identified as top contributors to sugar and fat intake indicating breastfeeding and complementary feeding education should be included as intervention components.

While the use of single 24-hour dietary recalls does not capture usual nutrient intake, single 24-hour recalls provide a snapshot of the study population’s current nutrition situation. Comparable studies using single 24-hour dietary recalls have also found that infants exceeded recommended levels for all nutrients, while toddlers 12–24 months of age failed to meet recommendations for vitamin E and fiber [14,33]. The observation of nutrient inadequacy among older infants and toddlers may have been due to low breast milk intake (which was excluded from ten most frequently reported foods) or the discontinuation of formula feeding. Inappropriate food choices by caregivers have also been observed in previous studies [34,35].

The accurate assessment of food intakes in children is challenging [1]. While parents tend to reliably report their children’s food intake in the home setting [2], interviews with caregivers in childcare settings could potentially be subject to bias due to low levels of interest and motivation [3]. As using multiple 24-hour recalls is the most reliable method of dietary assessment for preschool children [4], the information from a single day may not accurately reflect the usual diet of an individual [5]. Limitations of the study include the use of a single 24-hour recall to obtain dietary intake and the small sample size. While multiple 24-hour dietary recalls or weighed food records are ideal for obtaining nutrient intake data [36] the main purposes of this study was to obtain baseline dietary data, develop an appropriate food list, and identify the need for a nutritional intervention program. The relatively small sample population limits the generalizability of the results; however, the proposed nutrition intervention will target infants and toddlers residing in Baltimore. Although breast milk consumption was estimated based on established literature, this study contributes substantially to the limited literature on nutrient intake and dietary quality of infants and toddlers in Baltimore, MD.

## Conclusions

Infants were formula fed with a higher frequency than they were breastfed. The consumption of high-sugar and high-fat foods (e.g. sweetened drinks, French fries) increased with each age group, which can increase the risk of childhood obesity. The data were used to inform the Growing Leaps and Bounds (GLB) program, which promotes early obesity prevention among Baltimore infants and toddlers.

## Competing interests

The authors declare they have no competing interests.

## Authors’ contributions

SS conceptualized and participated in manuscript drafting, LB and NB participated in data collection and interpreted results, FK conducted the data analysis and finalized the manuscript, BR and GLM participated in data collection, JG and BC participated in manuscript drafting and provided a critical review. All authors read and approved the final manuscript.

## Supplementary Material

Additional file 1 **Food and drink items listed on the final food list.**^1^ Click here for file

Additional file 2 Sample page of FFQ. Click here for file
