# Supplementary material for: Assessing dietary intake among infants and toddlers 0–24 months of age in Baltimore, Maryland, USA
Source: Nutr J. 2013 Apr 26;12:52. doi: 10.1186/1475-2891-12-52 (PMC3644278; doi:10.1186/1475-2891-12-52)
Supplement: Additional file 1 — Food and drink items listed on the final food list.1 [file 1475-2891-12-52-S1.docx]

**Appendix I:** Food and drink items listed on the final food list^1^

| **Category (number of items)** | **Food items** |
| --- | --- |
| Breast milk and formula (2) | Any kind of infant formula (including lactose-free); breast milk |
| Dairy products (8) | Whole milk (including flavored, milk-shake, and lactose-free); 2%, 1% or fat-free (skim) milk (including flavored, milk-shake, and lactose-free); smoothie; yogurt (including drinkables); cottage cheese; any eggs (such as scrambled, fried, boiled); any hard cheese (such as American, Mozzarella sticks, cheddar); cream cheese and any soft cheese |
| Drinks (10) | Water (including bottled water); baby juice diluted with water; baby juice; juice drinks (not 100% juice); 100% juice diluted with water; 100% juice; sweetened tea; unsweetened tea; regular (non-diet) soda; frozen drink (Icee, Slurpee, or Snowball) |
| Cereals (5) | Any baby food cereal including rice, multigrain, oatmeal, or fruit puffs; baby cereal bar, baby granola bar or baby breakfast bar; unsweetened non-baby hot or cold cereal; sweetened non-baby hot or cold cereal; non-baby cereal bar |
| Fruits (15) | Any jarred pureed fruit baby food; applesauce; canned fruit in juice; canned fruit in syrup; fruit salad; apples or pears; bananas; cherries, green or red grapes; citrus fruits (e.g. oranges, tangerine); apricots, plums, or peaches; strawberries or other berries; kiwi; pineapple; dried fruit, including freeze dried; melon/cantaloupe |
| Baby food - vegetables and main dishes (4) | Any pureed vegetable jarred baby food; any jarred macaroni & cheese baby food; any jarred baby food chicken noodle soup; any jarred chicken or meat with pasta and/or vegetables baby food |
| Vegetables (not including those in soup/or stew) (16) | Mashed potatoes, potato salad, fried potatoes (hash browns, french fries, tater tots); sweet potato not including pie or yams; coleslaw; any corn; any mixed vegetables, green beans, string beans, or peas; any broccoli or cauliflower; any carrots eaten alone; any cabbage or greens; any squash or pumpkin not including pie; cucumber; tomato;lettuce; any lentils, beans, black-eyed peas, or chickpeas (including hummus); any tofu |
| Rice, pasta, and pizza (12) | Plain rice; not plain rice (cooked with broth or gravy); couscous; plain pasta with no added sauce (including oodles of noodles and orzo); oodles of noodles (including Ramen, cup of noodles); macaroni & cheese, pasta in cream sauce or pasta salad; spaghetti/noodles/pasta with meat sauce; spaghetti/noodles/pasta with tomato sauce (no meat); pizza; creamed soup; non-creamed soup |
| Meats (13) | Baked chicken or turkey (including wings); fried chicken, nuggets or strips, fingers, wings; chicken with sauce and vegetables (including Chinese); baked or fried pork; bacon; hot dog, scrapple, or sausage; ham or bologna; hamburger or meatloaf; any cut of beef, including roast beef; beef stew; fish, including tuna; seafood including crab or shrimp; quesadilla or fajita |
| Breads and crackers (8) | Non-sweetened crackers such as Saltines or those with peanut-butter filling; white bread, including bagels, English muffin, pita or toast; whole grain bread, including bagels, English muffin, pita or toast; plain tortilla; jelly/jam; peanut butter; butter/margarine on bread or crackers; waffles or pancakes |
| Snacks and desserts (11) | Baby food cookies or biscuits (including teething biscuits); cookies such as animal crackers, Oreo, rice crispie treats; chips, corn puffs, pretzels, popcorn; candy (hard or soft) including chocolate; fruit snacks; popsicle; gelatin/jello, pudding (such as banana or rice pudding); cakes or cupcakes (such as brownies or muffins); pastries (including donuts); fruit pie (such as pumpkin or sweet potato pie); ice cream/ice cream sundae/ice cream cake/ice cream sandwich |
| Supplement (1) | Multi-vitamins, such as Flintstones or gummy vitamins |
| Feeding practices (5) | Do you usually add cereal to the bottle or cup? ; Do you usually add milk to oatmeal or other hot cereal? ; Do you usually add sugar to the cereal? ; Is your pasta usually whole-wheat? ; Do you add butter/margarine to xx’s rice, pasta, or vegetables? |

^1^Supplement use and feeding practices also included
