# Supplementary material for: Assessing dietary intake among infants and toddlers 0–24 months of age in Baltimore, Maryland, USA
Source: Nutr J. 2013 Apr 26;12:52. doi: 10.1186/1475-2891-12-52 (PMC3644278; doi:10.1186/1475-2891-12-52)
Supplement: Additional file 2 — Sample page of FFQ. [file 1475-2891-12-52-S2.docx]

**Appendix II: Sample page of FFQ**

**Infant ID __ __ __**

| **How often during the past 30 days did XX have the following foods?** | Never | 1/mo | 2-3/m | 1/wk | 2-3/wk | 4-6/wk | 1/d | 2-4/d | 5+/d |
| --- | --- | --- | --- | --- | --- | --- | --- | --- | --- |
| **Breast milk and formula** |  |  |  |  |  |  |  |  |  |
| Any kind of infant formula (including lactose-free) | 1 | 2 | 3 | 4 | 5 | 6 | 7 | 8 | 9 |
| Breast milk | 1 | 2 | 3 | 4 | 5 | 6 | 7 | 8 | 9 |
| Do you usually add cereal to the bottle or cup?  Yes 1 No 2 N/A 3 |  |  |  |  |  |  |  |  |  |
| **Dairy products** |  |  |  |  |  |  |  |  |  |
| Whole milk (including flavored, milk-shake, and lactose-free) | 1 | 2 | 3 | 4 | 5 | 6 | 7 | 8 | 9 |
| 2%, 1%, or fat-free (skim) milk (including flavored, milk-shake, and lactose-free) | 1 | 2 | 3 | 4 | 5 | 6 | 7 | 8 | 9 |
| Smoothie |  |  |  |  |  |  |  |  |  |
| Yogurt (including drinkable) | 1 | 2 | 3 | 4 | 5 | 6 | 7 | 8 | 9 |
| Cottage-cheese | 1 | 2 | 3 | 4 | 5 | 6 | 7 | 8 | 9 |
| Any eggs (such as scrambled, fried, boiled) | 1 | 2 | 3 | 4 | 5 | 6 | 7 | 8 | 9 |
| Any hard-cheese (such as American, Mozzarella Sticks, Cheddar) | 1 | 2 | 3 | 4 | 5 | 6 | 7 | 8 | 9 |
| Cream-cheese and any soft cheese (such as cheese spread) | 1 | 2 | 3 | 4 | 5 | 6 | 7 | 8 | 9 |
|  |  |  |  |  |  |  |  |  |  |
| **Drinks** |  |  |  |  |  |  |  |  |  |
| Water alone, including bottled water | 1 | 2 | 3 | 4 | 5 | 6 | 7 | 8 | 9 |
| Baby juice **diluted with water** | 1 | 2 | 3 | 4 | 5 | 6 | 7 | 8 | 9 |
| Baby juice | 1 | 2 | 3 | 4 | 5 | 6 | 7 | 8 | 9 |
| Drinks that are not 100% juice, such as Sunny Delight, kool aid, gatorade, lemonade | 1 | 2 | 3 | 4 | 5 | 6 | 7 | 8 | 9 |
| 100% juice **diluted with water** (such as Juicy Juice w/water) | 1 | 2 | 3 | 4 | 5 | 6 | 7 | 8 | 9 |
| Drinks that are 100% juice, such as Juicy Juice | 1 | 2 | 3 | 4 | 5 | 6 | 7 | 8 | 9 |
| Sweetened tea | 1 | 2 | 3 | 4 | 5 | 6 | 7 | 8 | 9 |
| Unsweetened tea | 1 | 2 | 3 | 4 | 5 | 6 | 7 | 8 | 9 |
| Regular (non-diet) soda | 1 | 2 | 3 | 4 | 5 | 6 | 7 | 8 | 9 |
| Frozen drink, such as Icee, Slurpee, or Snowball | 1 | 2 | 3 | 4 | 5 | 6 | 7 | 8 | 9 |
|  |  |  |  |  |  |  |  |  |  |
| **Cereals** |  |  |  |  |  |  |  |  |  |
| Any baby food cereal including rice, multigrain, oatmeal, or fruit puffs | 1 | 2 | 3 | 4 | 5 | 6 | 7 | 8 | 9 |
| Any baby cereal bar, baby granola bar or baby breakfast bar (Gerber) | 1 | 2 | 3 | 4 | 5 | 6 | 7 | 8 | 9 |
